# Prevotella bivia: A Rare Cause of Zuska’s Breast Disease

**DOI:** 10.7759/cureus.28904

**Published:** 2022-09-07

**Authors:** Ameer Aboud, Andrew Smith, Shamon Gumbs, Alexius Ramcharan

**Affiliations:** 1 General Surgery, Columbia University College of Physicians and Surgeons, Harlem Hospital Center, New York, USA

**Keywords:** prevotella bivia, nonpuerperal breast abscess, recurrent subareolar abscess, lactiferous fistula, lactiferous sinus, zuska's disease, subareolar abscess

## Abstract

Zuska’s breast disease is a rare disorder leading to recurring subareolar abscess typically in obese patients with a history of smoking. The pathophysiology is a combination of ductal obstruction from squamous metaplasia and infection usually by anaerobic and gram-positive bacteria. Zuska’s breast disease does not have a single standardized treatment partly attributed to the lack of physician awareness of the disorder. The initial management should include smoking cessation, anaerobic and gram-positive antibiotic coverage, and drainage of abscesses if present.

## Introduction

Chronic, recurrent subareolar breast abscess, or Zuska’s breast disease, is a distinct entity of nonpuerperal breast abscess. Due to the rarity and lack of physician awareness of this condition, many patients are plagued with high recurrence rates without adequate understanding or explanation of their condition. This results in poor follow-up with a single provider, and subsequently patients have multiple visits to various clinics, urgent care centers, and emergency rooms in seek of a cure. Due to the recurring nature of the disease, many patients will receive multiple procedures on the breast, risking disfiguration and scarring of the breast.

We are reporting a rare case of Zuska’s breast disease caused by *Prevotella bivia* in an attempt to increase provider awareness of such cases. Therapeutic regimens and a review of the literature will also be discussed. To our knowledge, this is the only documented case of nonpuerperal breast abscess caused by *Prevotella bivia* in the United States of America [1].

## Case presentation

Our patient is a 56-year-old African American female suffering from obesity, hypertension, and hyperlipidemia with a history of bilateral breast abscesses requiring incision and drainage (I&D). She is a former smoker who reports quitting recently. She presented to the emergency department (ED) with a 10-day history of left breast pain, tenderness, and erythema. She had taken cefalexin for several days before the presentation with no improvement.

The patient was in no acute distress, afebrile, and tachycardic to low 100s at presentation. She had an erythematous, tender, fluctuant mass to the 12 o’clock position of the left areolar border with surrounding induration. There was nipple inversion present bilaterally. There were no palpable lymph nodes in either axilla. She had leukocytosis to 14,000, with the remainder of labs grossly normal. An I&D was performed in the operating room and 10 ml of purulent fluid was evacuated. The cavity was irrigated with saline and hydrogen peroxide, and the wound was left open and packed with iodoform gauze strips. She was started on IV clindamycin 600 mg preoperatively, followed by oral clindamycin 300 mg every 8 hours for ten days upon discharge. Intraoperative cultures revealed moderate *Prevotella bivia*, and no susceptibility was performed by the laboratory at that time. She was discharged postoperatively with visiting nurse services, however, she had not returned to the clinic as scheduled and was lost to follow-up.

Ten months later the patient presents to our emergency department reporting persistent drainage and tenderness from the same incision site performed initially. She had been seen at multiple urgent care centers and had taken numerous courses of antibiotics, whose names she could not recall. On examination, the left breast had a 2 x 2 mm, erythematous fistula to the 12 o’clock position at the areolar border, the site of the previous incision. There was a scant amount of purulent drainage, and bedside ultrasound did not reveal any evidence of collection. She was discharged from the emergency department and prescribed symptomatic therapy, a formal breast ultrasound, and sulfamethoxazole-trimethoprim (800-160 mg) every 12 hours for ten days.

Again, she did not follow up as scheduled, and presented to the clinic one month later reporting improved symptoms, but persistent drainage from the fistula. She was prescribed a repeat course of sulfamethoxazole-trimethoprim (800-160 mg) every 12 hours for ten days. Bilateral breast ultrasound and diagnostic mammogram were ordered to rule out any drainable collection, and also to rule out an inflammatory carcinoma. The imaging revealed only heterogeneously dense breast tissue, which is a benign finding.

**Figure 1:**
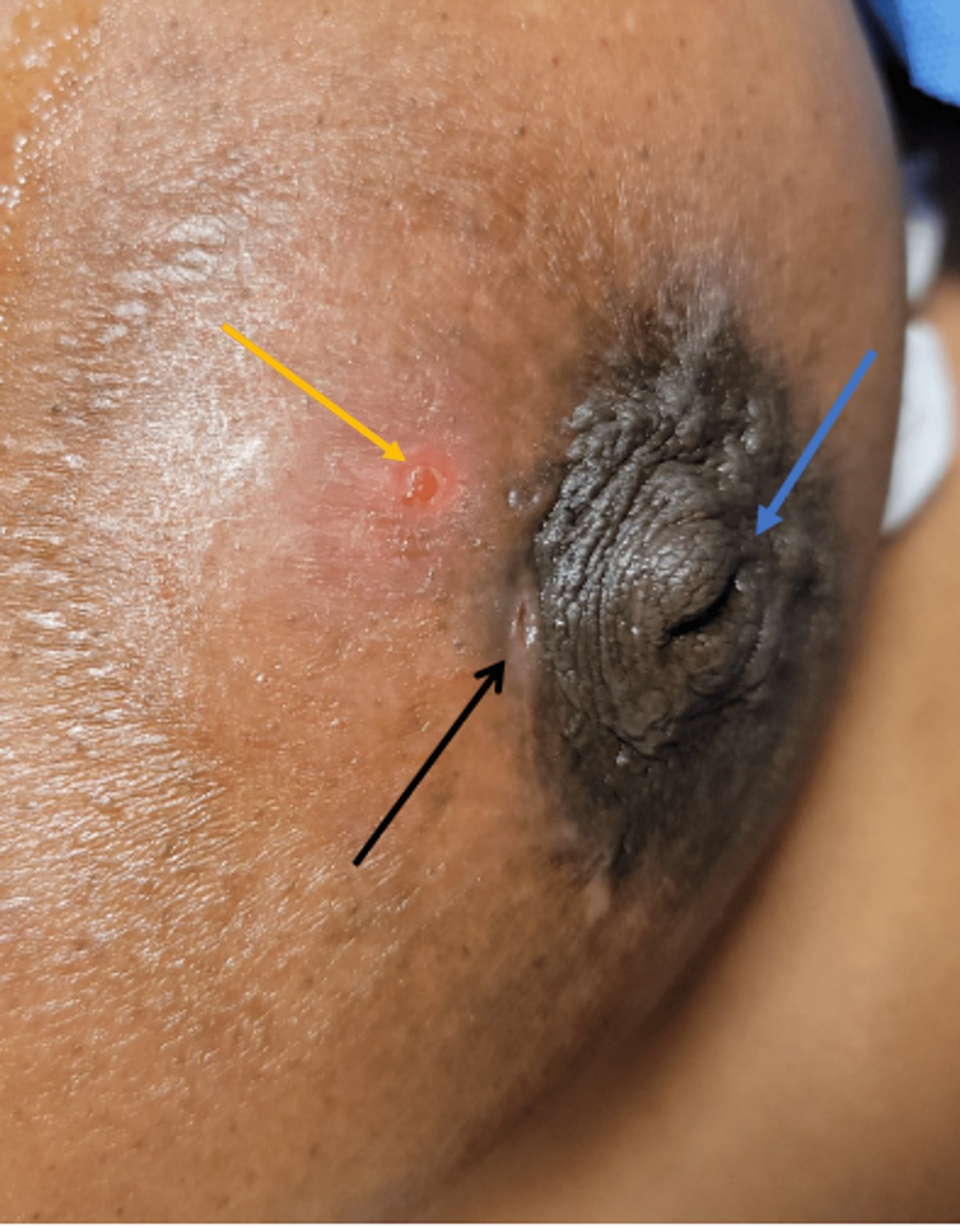
Subareolar breast abscess with periareolar fistula The black arrow demonstrates the periareolar draining fistula. The yellow arrow demonstrates an area of skin thinning over an underlying abscess. The blue arrow demonstrates prominent nipple inversion.

One month later, she presented to the emergency department with increasing pain and swelling to the left breast in the location of the previous fistula. There was localized erythema and tenderness to the left breast at the nipple-areolar complex (NAC). The previous fistula at the vermillion border of the areola persisted, as seen in the image below (Figure 1). Labs revealed a white blood cell count of 14.64. Point of care ultrasound revealed a small collection 1 cm deep to the areolar border. Bedside needle aspiration was performed, and 1-2 milliliters of purulent material was aspirated. She was discharged on clindamycin 300 mg every 6 hours for ten days and advised to follow up as an outpatient in the breast clinic.

Two weeks later she presents to the clinic with improvement of the inflamed breast, however, continued to have drainage from the periareolar fistula despite the completion of antibiotics. At this point surgical options were discussed with the patient, who refused any further invasive intervention. She was discharged on amoxicillin-clavulanate 875-125 mg every 12 hours and metronidazole 500 mg every 8 hours for ten days. At her next follow-up appointment after finishing this regimen, she had resolution of symptoms and finally healing of the fistulous tract.

## Discussion

Breast abscesses may be puerperal (lactational), or non-puerperal (non-lactational). A majority of breast abscesses are puerperal, occurring as a complication of mastitis. While puerperal abscesses are usually caused by skin flora, non-puerperal breast abscesses are often caused by anaerobic bacteria, in addition to *Staphylococcus *and *Streptococcus *species. Polymicrobial aerobic-anaerobic infections are also frequent [1,2].

Puerperal breast abscesses are common amongst lactating women, which form from bacterial proliferation within lactiferous ducts where there is milk stasis from infrequent emptying or microtrauma to the nipple occurring during feeding. Nonpuerperal breast abscesses are uncommon and are associated with smoking, obesity, diabetes, and trauma to the breast. The proposed pathogenesis includes periductal mastitis, follicular obstruction of the pilosebaceous unit, and infection arising in a cyst. Importantly, inflammatory carcinoma may also present initially as a nonpuerperal breast abscess [1,2].

Zuska et al. were the first to describe the association between the nonpuerperal subareolar abscess and lactiferous duct fistulas [3]. Zuska’s breast disease, also known as a recurring subareolar abscess, is a form of nonpuerperal breast abscess. The disease is characterized by a triad of a draining cutaneous fistula from the subareolar tissue, chronic, pasty discharge from the nipple, and a history of multiple, recurrent mammary abscesses [4]. These subareolar abscesses are postulated to occur mostly in patients with a history of smoking due to squamous metaplasia of the epithelial lining of the lactiferous ducts. Keratin debris produced may eventually lead to ductal obstruction. Once obstructed, infection ensues, and the duct may eventually rupture into the surrounding stroma, resulting in a chronic, active inflammatory reaction and abscess formation under the nipple. The occlusion of the lactiferous duct does not allow drainage to occur, causing frequent recurrence, and a fistulous tract may develop which typically opens at the border of the areola, as was seen in our patient [5].

Zuska’s breast disease is plagued with high recurrence rates. The periareolar fistula is initially lined by granulation tissue, but is eventually lined by keratinized epithelium which inhibits spontaneous healing. The presence of cellular debris in the mammary stroma with associated microbial growth results in chronic inflammation and discharge from the fistulous tract [5]. The usual pathogens associated with nonpuerperal breast abscess as mentioned before, include *Staphylococcus *and *Streptococcus *species, but a significant proportion are also caused by anaerobic bacteria such as *Bacteroides *species. In our patient, her cultures revealed growth of *Prevotella bivia*, which is an exceedingly rare cause of breast abscess, with only one other case report in the literature to our knowledge [1].

*Prevotella bivia *is an anaerobic, gram-negative rod, commonly found as normal flora in the oral cavity, gastrointestinal tract, and female genitourinary tract. Rare infections associated with *Prevotella* species include abscesses of the head and neck, otitis media, sinusitis, aspiration pneumonia, female genital tract infections, and intraabdominal infections [6]. *Prevotella bivia* is a rare cause of infection in the literature, with only 18 documented cases of infection globally, and the optimal antibiotic regimen reported from those cases varies widely [7]. Many of the cases were treated with multiple courses of different antibiotics, demonstrating the difficulty of properly treating infections caused by *Prevotella bivia*. Also, many of these were co-infections, with other organisms isolated in addition to *Prevotella bivia*. We would like to report a successful resolution of this rare condition with a course of amoxicillin-clavulanate and metronidazole, along with I&D and needle aspiration as needed for the presence of an abscess. This antibiotic regimen was reported to be successful in the only single similar case report of a nonpuerperal breast abscess caused by *Prevotella bivia* present in the literature [1].

A common error in the management of nonpuerperal breast abscess is treatment with narrow-spectrum gram-positive antibiotics. This strategy excludes anaerobes, which contribute to a significant proportion of nonpuerperal breast abscesses, and may be a contributing factor in the high recurrence rates noted with such abscesses [7]. Consideration should be made for treatment with broad-spectrum antibiotics which target anaerobic and gram-positive bacteria, such as amoxicillin-clavulanate, or clindamycin. Metronidazole may be added in the event of inadequate therapeutic response, as was seen in our case, after which we achieved a complete therapeutic response.

The management of recurrent, fistulous, subareolar abscesses is not well standardized and there is much debate in the current literature. A simple puerperal abscess is often treated successfully with antibiotics and drainage of abscess via needle aspiration or incision. In contrast, nonpuerperal breast abscess when treated similarly will commonly lead to recurrence, up to 79% in some case series [8]. The reason for such high rates of recurrence is unclear, and may be related to inadequate selection of antibiotic therapies, or failure to address the underlying mechanism by which nonpuerperal breast abscesses are thought to occur; ductal obstruction.

In our case, we hypothesize that an atypical infection with *Prevotella bivia*, believed to be resistant to many standard antibiotic regimens, resulted in inadequate treatment, contributing to the multiple recurrences she experienced. Combined with the lack of consistent follow-up to a single provider, and refusal of invasive surgical procedures, our patient ultimately suffered with this condition for a prolonged period of time. Fortunately, she did not experience any gross adverse events from the multiple courses of antibiotics. It is also unclear whether or not surgical resection would have been required for her earlier in her course, as ultimately the patient did have a complete response, albeit to a particular antimicrobial regimen.

Some authors have reported successful conservative treatment of nonpuerperal breast abscess with appropriate antibiotics and needle aspiration as needed, and reserve surgical management for cases in which medical management fails [9]. Other authors advocate for early surgical intervention with excision of the initiating process, which is the large, distended, or occluded lactiferous duct in addition to the chronically infected tissue; the fistula tract, and abscess. These authors report lower recurrence rates amongst patients whose treatment included excision of the involved lactiferous ducts (28%) than among those whose treatment did not (79%) [8].

Various surgical techniques have been described in the literature. Meguid et al. described a transverse incision from the nipple (to include the diseased duct) laterally through the areola to the vermillion border with limited dissection of the diseased duct, and partial re-approximation of the anatomic borders of the nipple areolar-complex. The wound was then packed to heal by secondary intent [10].

A radial technique is described by Lannin, in which an ellipse of tissue was excised, allowing a wide resection to normal tissues in a “slice of pie” fashion, whereby afterward the wound would be closed primarily with acceptable cosmesis [11]. In this case series, it was concluded that approximately half of the patients with nonpuerperal subareolar abscess would resolve with needle/incisional drainage and antibiotics, while the other half required ductal excision. Recurrence of the abscess or fistula formation were significant factors predicting the need for ductal excision.

## Conclusions

Zuska’s breast disease is a rare cause of considerable morbidity, plagued with recurrences due to underdiagnosis and inadequate treatment. With an increase in provider awareness of this condition and its natural history, a timely diagnosis can be made, expediting treatment. Therapeutic options should include initial smoking cessation, drainage of abscess, appropriate antimicrobial coverage, and if necessary, surgical excision of the offending duct with surrounding inflammatory tissue. Cultures should be obtained, especially in recalcitrant cases, as in our case, rare pathogens such as *Prevotella bivia* may be a source of prolonged morbidity. Prevention of this condition includes avoidance of tobacco, weight loss, and appropriate glucose control. Further studies are required to determine the optimal management strategy and indications for surgical intervention for this rare condition.
